# Effects of 12-Week Low or Moderate Dietary Acid Intake on Acid–Base Status and Kidney Function at Rest and during Submaximal Cycling

**DOI:** 10.3390/nu10030323

**Published:** 2018-03-08

**Authors:** Enni-Maria Hietavala, Johanna K. Ihalainen, Lynda A. Frassetto, Moritz Schumann, Daniela Eklund, Hannu Pitkänen, Keijo Häkkinen, Antti A. Mero

**Affiliations:** 1 Biology of Physical Activity, Faculty of Sport and Health Sciences, University of Jyväskylä, P.O. Box 35 (VIV), 40014 Jyväskylä, Finland; johanna.k.ihalainen@jyu.fi (J.K.I.); daniela.eklund@gmail.com (D.E.); keijo.hakkinen@jyu.fi (K.H.); antti.a.mero@jyu.fi (A.A.M.); 2 General Clinical Research Center, University of California San Francisco, 505 Parnassus Avenue, San Francisco, CA 94117, USA; Lynda.Frassetto@ucsf.edu; 3 Department of Molecular and Cellular Sports Medicine, German Sport University, Am Sportpark Müngersdorf 6, 50933 Cologne, Germany; m.schumann@dshs-koeln.de; 4 Honka Holding, c/o Honkatarhat Oy, Kirkkokallio 20, 38950 Honkajoki, Finland; hannu.pitkanen@mykora.com

**Keywords:** dietary acid load, acid–base status, net acid excretion, exercise training, kidney function

## Abstract

Prolonged effects of dietary acid intake on acid–base status and kidney function have not yet been studied in an intervention study in healthy subjects. Dietary acid load can be estimated by calculating the potential renal acid load (PRAL) of foods. Effects of low-PRAL and moderate-PRAL diets on acid–base status and kidney function were investigated during a 12-week exercise training period. Healthy, 20–50-year-old men (*n* = 21) and women (*n* = 25) participated in the study and were randomly divided into low-PRAL and moderate-PRAL groups. Before (PRE), mid-phase (MID) and after the intervention (POST), the subjects participated in measurement sessions, where a 12-h urine sample and fasting blood samples were collected, and a submaximal cycle ergometer test was performed. Net acid excretion was significantly lower after 12 weeks of the low-PRAL diet as compared to the moderate-PRAL diet, both in men and women. In low-PRAL females, capillary pH and bicarbonate were significantly higher at 75% of VO_2max_ at POST as compared to PRE. Glomerular filtration rate decreased over the study period in moderate-PRAL men and women. The results of the present study suggest that an acidogenic diet and regularly training together may increase the acidic load of the body and start to impair the kidney function in recreationally active subjects.

## 1. Introduction

Many biochemical reactions release and bind hydrogen ions (H^+^) in the human body. Under normal physiological conditions, diet composition is the primary modifier of net endogenous acid production (NEAP) and it may further affect the acid–base status of the body [1]. Dietary acid load can be estimated by calculating the potential renal acid load (PRAL) of foods, which estimates the acidic potential of foodstuffs [2]. Digestion of large amounts of animal protein and grain products, but only small amounts of vegetables and fruits, leads to a net production of acids in the body [3]. H^+^ concentrations in body fluids are regulated to remain in between rather narrow pH limits and thus, only minor changes occur in blood pH. In arterial blood at rest, pH is normally maintained strictly between 7.35–7.45 and extracellular buffering (i.e., mainly bicarbonate buffering) occurs concomitantly with any changes in plasma H^+^ concentration. However, it has been shown that diet composition may cause acute changes inside the optimal blood pH range [4]. Urine pH can vary between 4.5 and 8.0, according to the amount of H^+^ that needs to be excreted from the body by the kidneys. The systemic bicarbonate (HCO_3_^−^) concentration represents the metabolic component of acid–base balance, and the kidneys have a prevalent role in regulating it [5]. 

In the field of exercise physiology, effects of exercise on kidney function have not been studied very intensively. The kidney has an essential role in the homeostasis of the body at rest, but changes in renal function also occur with exercise. Under resting conditions, blood flow to the kidneys is among the highest to any organ. However, oxygen consumption is not increased in renal tissue during exercise, and blood flow is redistributed away from the kidney to skeletal muscles [6]. The glomerular filtration rate (GFR) is a measure of the amount of fluid filtered through the glomerular basement membrane in the kidneys, and it is considered to be the best overall assessment of kidney function [7]. With exercise loads up to 50% of VO_2max_, GFR is slightly increased or unchanged, and at higher exercise intensities, GFR decreases at higher rates than renal blood flow [8]. The long-term effects of exercise training on kidney function have not been studied in healthy populations, but in patients suffering decreased kidney function there is evidence for an association between kidney function and exercise performance [9]. Moreover, in a study by Morales et al. [10], kidney function affected the physical performance of athletes, as VO_2max_ was lower and heart rate higher in a group of athletes with smaller GFR, compared to athletes with higher GFR. It was recently shown that dietary acid load may play a role in delaying fatigue during exercise as the effects of dietary acid load on acid–base status and physical performance were investigated during a 7-day diet period [11]. The data suggested that lower acid intake could help the kidneys to increase exercise capacity by maintaining a higher extracellular HCO_3_^−^ concentration, which could delay the onset of fatigue caused by exercise-induced acidosis.

To the best of our knowledge, the prolonged effects of dietary acid intake on acid–base status and kidney function, with or without exercise training, have not yet been studied in an intervention study in healthy subjects. The aim of the present research was to study how dietary acid load affects the acid–base status of the body during a 12-week combined endurance and strength training period at rest and during submaximal cycling. In addition, the effects of a diet and training intervention on kidney function were investigated at rest. The kidney function was assessed with GFR, serum urea-to-creatinine ratio (UCR) and serum urea. It was hypothesized that the lower acid intake would induce a less acidic blood acid–base status—that is, higher pH and HCO_3_^−^—at rest and during submaximal exercise and would preserve kidney function at rest.

## 2. Materials and Methods

### 2.1. Subjects

In total, 49 healthy men and women volunteered and were selected to participate in the study. The study participants were required to be 20–50 years old and recreationally physically active. Before inclusion into the study, the physical activity of the subjects was characterized by walking, cycling, team sports, or strength training at a light-to-moderate intensity, at a frequency of 1–3 times per week, but a lack of systematic engagement in any endurance or strength training. The female participants were allowed to use contraceptive pills during the study period, but any other medication was considered to be exclusion criteria. Also subjects whose body mass indexes were above 33 kg/m^2^ or who had any relevant food allergy were excluded from the study. Ethical approval was obtained from the Ethical Committee of the University of Jyväskylä, and the study was in accordance with the Helsinki Declaration. Prior to the first testing, subjects were informed of the purpose and the methods of the study, and they signed a written informed consent. Additionally, the subjects completed questionnaires about their health, diet, and exercise background, and underwent a standardized resting electrocardiogram procedure, which was reviewed by a cardiologist. At the beginning of the study, the subjects were randomly divided into the low-PRAL and the moderate-PRAL diet groups and ate accordingly for the entire duration of the study period. Over the study period, there were three drop-outs, for reasons unrelated to the intervention. Baseline anthropometric characteristics of the subjects who completed the entire data collection are presented in Table 1.

### 2.2. Study Design

The study period lasted for 12 weeks. The subjects trained twice a week, and every training session consisted of both endurance and strength training (approximately 45 min + 45 min each). The measurement sessions took place before the intervention period (PRE), at the mid-phase during weeks 6–8 (MID) and after the intervention (POST). During each testing session a 12-h urine sample and fasting blood samples were collected. In addition, the subjects recorded their food intake via a 3-day food diary. At PRE and POST, the subjects also performed a submaximal cycle ergometer test during which the blood samples were obtained.

One week before the start of the 12-week intervention, the VO_2max_ and maximal workloads of the subjects were measured via an incremental cycle ergometer test that was performed on a microprocessorcontrolled, eddy current brake equipped ergometer (Ergoline ergometrics 800, D-72475, Bitz, Germany). The initial workload was 50 W and it was increased by 25 W every 2 min until volitional exhaustion occurred. VO_2max_ was determined to be the highest 30-s VO_2_ value during the test and coincided with at least one of the following two criteria: (a) respiratory exchange ratio >1.1; and/or (b) a plateau of oxygen uptake (less than 150 mL/min increase in VO_2_ during the last 60 s of the test). Gaseous exchange was measured using a Jaeger Oxycon Pro breath-by-breath gas analyzer (VIASYS Healthcare GmbH, Hoechburg, Germany). The device was calibrated for volume and gas before every measurement. The VO_2max_ determined at baseline (PRE) was used in all subject groups to set the workloads for the submaximal cycling tests. Three days after the VO_2max_ test, the subjects performed a submaximal cycling test (PRE) that started with a 5-min warm-up, followed by a 4-min break. Thereafter, the subjects completed three 8 min trials at 35%, 55% and 75% of their VO_2max_. All workloads were separated by 4-min rest periods, during which blood samples (CT35, CT55, CT75, respectively; CT = cycling test) were collected from a fingertip capillary and an antecubital vein.

During the last 12 h before the start of the dietary intervention, subjects had a 12-h overnight fast and, at the same time, collected a 12-h urine sample. The next morning, in a laboratory, fasting blood samples (FAST) were drawn from a fingertip capillary and an antecubital vein. The blood samples were obtained at 7–10 a.m. and kept similar throughout the study. The 12-h urine collection commenced 12 h before FAST. Starting from PRE, the subjects followed either the low-PRAL or the moderate-PRAL diet, and the same urine and blood sampling sessions were repeated at MID and at POST. At PRE and POST, after the blood sampling, the body composition of the subjects was assessed by dual X-ray absorptiometry (DXA) (Lunar Prodigy Advance, GE Medical Systems, Madison, WI, USA). Total fat mass and total lean mass were automatically analyzed (enCORE software, version 14.10.022, GE Medical Systems, Madison, WI, USA). Thereafter, the subjects ate a light breakfast, which was consistent with their assigned diet. Resting blood samples were drawn once more (REST) before a submaximal cycle ergometer test was completed.

### 2.3. Diets

Dietary acid load can be estimated by calculating the potential renal acid load (PRAL) of foods, which represents the renal net acid excretion caused by a foodstuff [2]. The diets used in the present study were designed with the PRAL calculations to have low and moderate acid loads. The aim was that the low-PRAL diet would enhance the production of alkaline compounds in the body (PRAL < 0), whereas the moderate-PRAL diet was aimed to slightly increase the production of acid compounds (PRAL > 0). The PRAL values were calculated as follows: PRAL (mEq/100 g) = 0.49 × protein (g/100 g) + 0.037 × phosphorous (mg/100 g) − 0.021 × potassium (mg/100 g) − 0.026 × magnesium (mg/100 g) − 0.013 × calcium (mg/100 g) [2]. The nutrient contents of the food were taken from the Finnish Food Composition Database (Fineli, Finnish National Institute of Health and Welfare). Before the start of the 12-week intervention period, the subjects followed their normal diet and kept food diaries for 3 days. Appropriate dietary counselling was given for both diet groups based on the baseline dietary analysis, and the subjects were given instructions on how to follow the low- and moderate-PRAL diets. Both groups controlled nutritional intake according to the general dietary guidelines, but in the low-PRAL group, the subjects were advised to increase the consumption of fruits and vegetables up to 800–1000 g. On the other hand, in the moderate-PRAL group, the subjects were advised to limit their intake of fruits and vegetables to 200–300 g.

The subjects kept food diaries for 3 days at PRE, MID, and POST. In addition, the subjects recorded their food intake during weeks 1–4 via a 3-day food diary, in order to check if the diet that the subjects were assigned was followed according to the instructions. The food diaries were analyzed for energy, protein, carbohydrate, fat, phosphorous, potassium, magnesium and calcium intake using Nutri-Flow software (Flow-Team Oy, Oulu, Finland). The average daily PRALs during the experimental diets were calculated according to the relevant dietary intake data.

### 2.4. Urine Sampling and Analysis

The subjects collected 12-h urine samples at PRE, MID and POST. Each urine sample was collected in a sterile container and refrigerated until subjects came to the laboratory and brought the container with them. Upon receipt, urine pH was determined by dipping a pH strip into the urine (Combur-7 Test urinalysis test strips; Cobas, Roche, Germany).

Urine electrolytes were analyzed by the direct ISE in vitro test (Ion Selective Microlyte Analyzer, Konelab 20 XTi; Kone Instruments, Espoo, Finland). Indirect Net acid excretion (NAE) was calculated as follows:

NAE (mEq/day) = (Cl^−^ + P_i_ + SO_4_^−^ + OA) − (Na^+^ + K^+^ + Ca^2+^ + Mg^2+^),

where SO_4_^−^ = 0.4875 × dietary protein intake (g)

OA (organic acids) = (BSA × 41)/1.73

where BSA (body surface area) = [(weight (kg) × height (m))/3600]^½^ [12].

### 2.5. Blood Sampling and Analysis

All fasting capillary and antecubital vein blood samples were drawn at the same time, in the morning, at all three sampling points. Li-heparinized whole blood samples (200 and 20 µL) from a fingertip capillary were analyzed immediately after sampling for pH, and HCO_3_^−^. The determination of pH was based on the principle of the ion selective electrode, whereas HCO_3_^−^ was determined computationally from pH and pCO_2_ values (GEM Premier 3000, Instrumentation Laboratory, Lexington, MA, USA). The blood samples from the antecubital vein were drawn in vacuum tubes, stored at room temperature for 30 min and centrifuged for 10 min at 3500 rpm (2100× *g*). The serum was separated, and creatinine and urea were analyzed by a KoneLab 20 XTi analyzer (Thermo Electron Corporation, Vantaa, Finland). Serum creatinine values were used to calculate the glomerular filtration rate (GFR) with the CKD-EPI equation [13]. Also, the serum urea to creatinine ratio (UCR) was calculated.

### 2.6. Training

The training protocol has been described elsewhere [14]. A combination of aerobic and resistance training has been proposed to be the most effective strategy for maintaining and/or improving physical fitness and health [15]. Briefly, the endurance training was conducted on a cycle ergometer and the training program included mostly steady-state cycling of low to moderate intensity (below and above the aerobic threshold). The duration of endurance cycling increased progressively from 30 to 50 min. During the last 4 weeks of the training period, the intensity of cycling also increased from the aerobic to the anaerobic threshold and then further, until subjects were completing maximal aerobic workloads. The resistance training program included exercises for all major muscle groups with a focus on the lower extremities. During the first two weeks, training was performed as a circuit using low intensities. Thereafter, protocols aiming for muscle hypertrophy and maximal and explosive strength were performed. During the second half of the study, both training volume and frequency were increased. The overall duration of the strength training sessions was 30–50 min.

### 2.7. Statistical Analysis

The main purpose of the present study was to determine if dietary acid intake has an effect on the primary outcome variable: acid–base status. NAE, capillary pH and capillary HCO_3_^−^ were analyzed to identify the possible differences in acid–base status. The secondary outcome of the study was to assess kidney function, measured by GFR, the serum urea-to-creatinine ratio (UCR) and serum urea. The effect of a 12-week intervention period on blood and urine variables, and variables of dietary intake analysis were examined by a two-way repeated measures analysis of variance (ANOVA). If a statistically significant difference was observed within one of the diet groups, or between groups, the comparison was continued with a suitable *t*-test. Data are presented as means ± SDs. The statistical difference was considered to be significant at the *p* < 0.05 level.

## 3. Results

### 3.1. Diets

Dietary intake data are presented in Table 2 and Table 3. There were no significant differences in energy or macronutrient intakes within or between the diet groups, except in moderate-PRAL men, as their energy intake was significantly decreased from PRE to MID (*p* = 0.027). In both men and women, PRAL was significantly lower (*p* ≤ 0.001 in all) and the intake of fruits and vegetables (IFV) were higher (*p* ≤ 0.017 in all) in low-PRAL compared to moderate-PRAL at MID and POST. In low-PRAL men and women, PRAL was significantly lower (*p* ≤ 0.005 in all) and IFV higher (*p* ≤ 0.06 in all) at MID and POST, compared to PRE. There were no significant differences in PRAL and IFV between MID and POST in any of the groups. 

### 3.2. Body Composition of the Subjects

There were no significant changes in body mass, total lean mass or fat% in either of the subject groups over the 12-week study period (Table 4).

### 3.3. Urine and Blood Acid-Base Status

**Figure 1:**
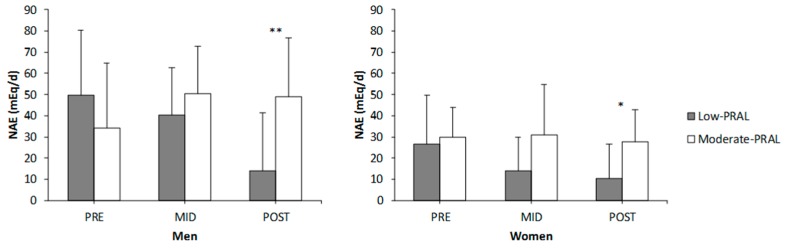
Net acid excretion (NAE) in the low-PRAL and the moderate-PRAL diet groups before (PRE), in the middle (MID) and after (POST) the 12-week diet period. * *p* < 0.05, ** *p* < 0.01 statistically significant difference between the diet groups at POST (two-way repeated measures ANOVA, an independent *t*-test).

NAE was lower in low-PRAL compared to moderate-PRAL at POST in both men (*p* = 0.001) and women (*p* = 0.047) (Figure 1). There were no statistically significant changes in urine pH, which was estimated with the pH test strips over the study period, in either of the subject groups. The urine strip results are not presented.

**Figure 2:**
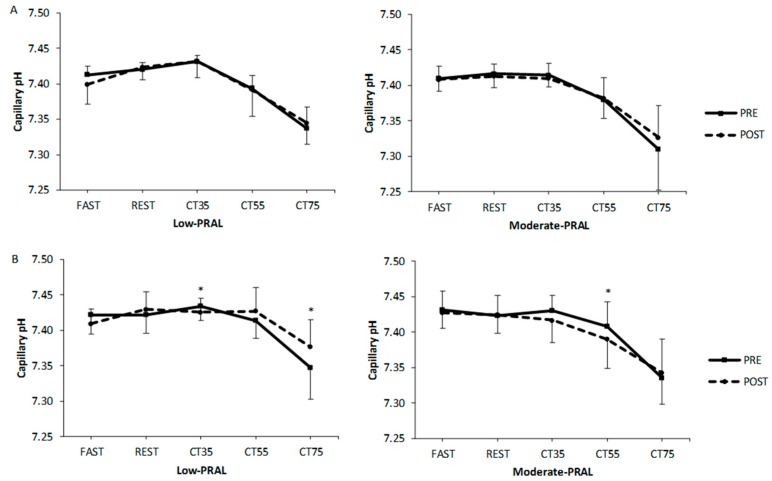
Capillary pH in men (**A**) and women (**B**) before (PRE) and after (POST) the 12-week diet period at rest (FAST, REST) and during submaximal cycling (CT35, CT55, CT75; CT = cycling test). * *p* < 0.05, statistically significant difference between PRE and POST within a diet group (two-way repeated measures ANOVA, a paired *t*-test). Values are mean ± SD.

For capillary pH, there were no significant differences between the diet groups (Figure 2). In low-PRAL women, pH was significantly lower (*p* = 0.014) at 35% and higher (*p* = 0.020) at 75% of VO_2max_ at POST compared to PRE. In moderate-PRAL women, pH was significantly lower at 55% (*p* = 0.033) of VO_2max_ at POST compared to PRE.

**Figure 3:**
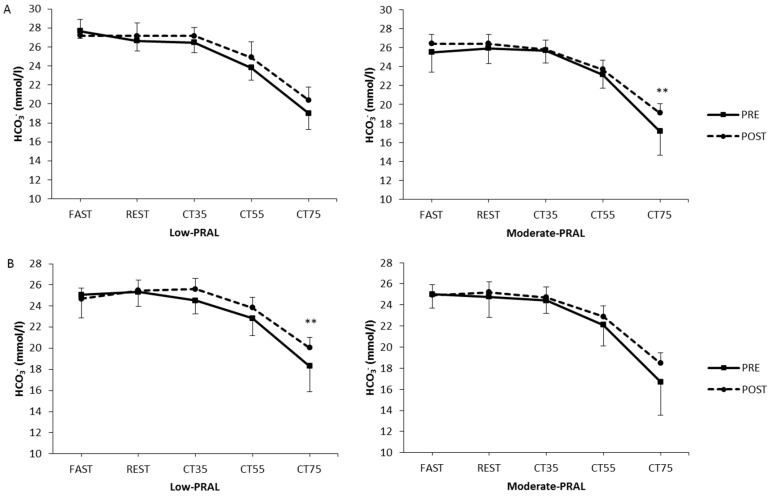
Capillary bicarbonate in men (**A**) and women (**B**) before (PRE) and after (POST) the 12-week diet period at rest (FAST, REST) and during submaximal cycling (CT35, CT55, CT75). ** *p* < 0.01, statistically significant difference between PRE and POST within a diet group (two-way repeated measures ANOVA, a paired *t*-test).

In low-PRAL women, HCO_3_^−^ was higher (*p* = 0.006) at 75% of VO_2max_ at POST compared to PRE (Figure 3). In moderate-PRAL men, HCO_3_^−^ was higher (*p* = 0.002) at 75% of VO_2max_ after the training period compared to PRE. The only significant differences in HCO_3_^−^ between the diet groups occurred in men, as HCO_3_^−^ was higher at FAST, REST and during cycling at 35% of VO_2max_ in low-PRAL compared to moderate-PRAL (*p* = 0.015, *p* = 0.039, *p* = 0.010, respectively). 

### 3.4. Renal Function

**Figure 4:**
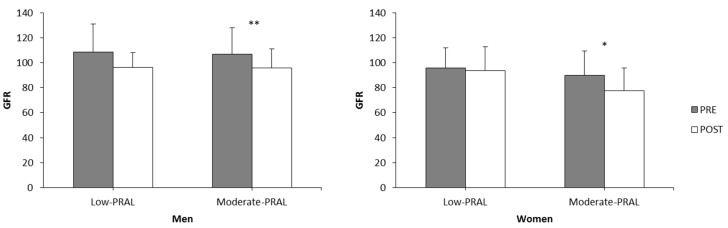
Glomerular filtration rate (GFR) in the low-PRAL and moderate-PRAL groups before (PRE) and after (POST) the 12-week diet period. * *p* < 0.05, ** *p* < 0.01 statistically significant difference between PRE and POST within a diet group (two-way repeated measures ANOVA, a paired *t*-test).

GFR decreased in the moderate-PRAL men (*p* = 0.009) and women (*p* = 0.036) over the dietary intervention (Figure 4). There were no significant changes in the low-PRAL groups over the study period.

**Figure 5:**
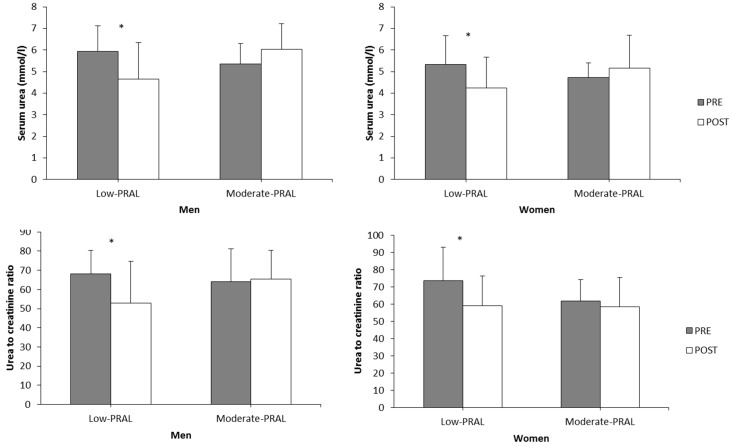
Serum urea and urea to creatinine ratio in the low-PRAL and moderate-PRAL groups before (PRE) and after (POST) the 12-week diet period. * *p* < 0.05 statistically significant difference between PRE and POST within a diet group (two-way repeated measures ANOVA, a paired *t*-test).

Serum urea decreased significantly in the low-PRAL men (*p* = 0.037) and women (*p* = 0.013) (Figure 5). Also, the serum urea to creatinine ratio decreased over the low-PRAL diet period in both men (*p* = 0.030) and women (*p* = 0.016) (Figure 5). No significant changes were observed in the moderate-PRAL diet groups for either of the variables.

## 4. Discussion

In the present study, recreationally active, healthy men and women followed either a low-PRAL or a moderate-PRAL diet for 12 weeks and participated in same session of combined endurance and strength training twice a week. Net acid excretion (NAE) was significantly lower after 12 weeks of lower dietary acid intake compared to higher acid intake, both in men and women. There were no significant differences in urine or capillary pH or capillary bicarbonate between the diet groups after the study period. However, in low-PRAL women, capillary pH and bicarbonate were significantly higher at 75% of VO_2max_, whereas in moderate-PRAL women, capillary pH was significantly lower at 55% of VO_2max_ after the study period compared to at baseline. According to the estimated glomerular filtration rate (GFR), kidney function was significantly decreased over the study period in both moderate-PRAL men and women. There was no change in serum urea or the urea-to-creatinine ratio (UCR) in the moderate-PRAL participants, whereas in the low-PRAL groups, these blood variables decreased over the 12-week intervention. These results suggest that even slightly acidogenic diets and regular training together may lead to an increased acid load to the body and start to impair kidney function in recreationally active subjects. However, these results should be interpreted with caution since there was some variation in energy intake and in the body composition, at least in the males, during the study period.

The only significant difference in the diet composition between the low-PRAL and moderate-PRAL groups was in the consumption of fruits and vegetables. This strengthens the fact that intake of fruits and vegetables is an important factor affecting dietary acid load and net acid excretion of the body. There were clear differences in NAE between the diet groups in both men and women. Urine pH was at higher level in low-PRAL as compared to moderate-PRAL, in both men and women, after the diet intervention, but the differences were not statistically significant. However, urine pH was not measured; rather, it was determined with test strips which provide only rough estimates of pH. It has been previously shown that urine and capillary pH can change acutely due to the dietary acid intake that has been estimated with PRAL [4]. There were no differences in capillary pH between the diet groups, but inside both female groups there were some differences during submaximal exercise between PRE and POST. It was observed that in low-PRAL women, capillary pH was higher at the two highest exercise intensities after the diet and training intervention, whereas in moderate-PRAL women, the blood pH was more acidic at the two lowest exercise intensities after the study period. These data increase the body of evidence showing that diet-induced changes in acid–base status are small yet viable. In spite of powerful regulatory mechanisms, which ensure that there do not appear to be large changes in acid–base balance, it seems that these regulatory mechanisms do not keep pH and HCO_3_^−^ at a static level; rather, some variations can occur, based on the daily diet- and exercise-induced acid loads that the body confronts. On the other hand, in the study of Wesson and Simoni [16] it was demonstrated that rats with lower kidney masses were not able to excrete the same acid load in their urine, while, at the same time, renal tissue acid levels were higher, with no differences in blood acid–base levels between the two groups. Our data supports the idea that even though NAE changes, it does not necessarily reflect the changes in urine and blood pH. It has been speculated that some of the increased H^+^ is bound predominantly to intracellular body buffers [17]. This would also contribute to the fact that women seem to be more sensitive to the changes in dietary acid load than men, who have larger muscle masses and thus, larger tissue buffering capacities. However, for those who have an intact renal functional capacity, constant intake of alkaline products might be beneficial by providing a greater reserve to buffer high acid loads. This might be important, for example, for exercise performance and, in particular, for the elderly, who have diminished renal functional capacities [11,18].

The moderate-PRAL diet of the present study represented a typical diet for many Westernized cultures; it contained animal protein and cereal grains and was quite deficient in vegetables and fruits—a type of a diet that may increase the acid load of the body [3]. Also exercise training acutely impacts the acid–base status and after the capacity of chemical buffers and ventilation is surpassed, the kidneys need to excrete acids, neutralize acids and/or excrete anions to maintain the acid–base balance [19]. In the present study, GFR decreased in the moderate-PRAL groups of both men and women over the 12-week intervention, which could be due to the increased acid load from both the diet and exercise training. There has been some debate about whether diet composition could affect renal function over a longer period of time. In particular, a high intake of protein has been suggested to potentially impair kidney function. In individuals with moderate to severe renal insufficiency, a low protein intake may slow renal function decline, but the long-term impact of protein intake on kidney function in individuals with normal kidney function or mild renal insufficiency is unknown [20]. A recent paper by Møller et al. [21] reported no association between ahigher protein intake and decreased kidney function in pre-diabetic older adults during a one-year intervention. In a study by Antonio et al. [22], high protein intake (2.5 g/kg/day) was compared to higher intake (~3.3 g/kg/day), and it was reported that these high protein diets were not deleterious for kidney function over a one-year crossover study period. However, in the present study, protein intake did not differ between the diet groups, suggesting that not only the protein intake, but also the dietary acid load, should be considered as factors that may affect renal function. This was also proposed by So et al. [23], who showed that dietary acid load might be a better indicator of the changes in renal function associated with the habitual dietary pattern than merely the total amount of dietary protein. In addition to the effects of dietary acid load on blood and urine pH, a high dietary acid intake has been reported to be associated with faster decline in kidney function in kidney patients, which deteriorates the capacity of the kidneys to handle acids and may further increase the acidity of the body [24]. The aging population is likely more prone to increased dietary acid loads, but the results of the present study suggest that reducing the acid load in younger, physically active populations could be beneficial for kidney function. At present, the very long-term effects of high acid load in healthy populations are not known.

When assessing kidney function via GFR, it has to be kept in mind that serum creatinine can be affected by dietary protein intake and the lean mass of the body [8]. One of the limitations of the present study is that the CKD-EPI equation used to evaluate GFR does not take into account the effect of muscle mass on serum creatinine, which is a breakdown product of creatine phosphate in the muscle. In the moderate-PRAL group, it seems that the lean mass was slightly increased over the 12-week intervention, which may have had an impact on serum creatinine and thus, on GFR. However, these changes were not significant and, furthermore, there was no correlation between serum creatinine and lean muscle mass in either of the subject groups. Nonetheless, serum urea and UCR were decreased in the low-PRAL group, and they remained unchanged in the moderate-PRAL group, both in men and women, even though there were no changes in protein intake within or between the diet groups. This supports the idea that the changes observed in GFR were not solely due to the changes in the lean mass of the subjects. On the other hand, it shows that markers that have been considered to be markers of protein intake and metabolism can change while the protein intake remains stable and highlights the importance of dietary acid load for kidney function. However, cystatin C, a more stable marker that is less affected by muscle mass, should be considered as a potential replacement for serum creatinine for assessing GFR in future studies [8]. Moreover, to confirm the results of the present study, the long-term effects of dietary acid load should be studied with larger sample sizes and with a longer duration, since 12 weeks is a short period of time to investigate changes in kidney function. In addition, one of the limitations of the present study was the decreased energy intake in the group of low-PRAL men over the study period.

In conclusion, 12-week low or moderate dietary acid intakes showed some varying effects on urine and blood acid–base status and kidney function in healthy, recreationally active men and women. NAE was significantly lower after 12 weeks of low dietary acid intake compared to moderate acid intake both in men and women, but the effect of a low dietary acid intake on blood acid–base status was apparent only for women. GFR decreased in the moderate-PRAL groups over the 12-week intervention, which suggests that a slightly acidogenic diet and regularly training together may increase the acid load of the body and start to impair kidney function. These results emphasize the importance of adequate intake of fruits and vegetables as a part of a healthy diet and a physically active lifestyle. In future studies, dietary acid intake should be taken into account when investigating the combined effects of diet and exercise on health. 

## Figures and Tables

**Table 1:** Baseline anthropometric characteristics of the subjects in the low-potential renal acid load (PRAL) and moderate-PRAL diet groups.

Parameters	Women		Men	
	Low-PRAL	Mod-PRAL	Low-PRAL	Mod-PRAL
***N***	13	12	9	12
**Age (years)**	34.3 ± 6.9	32.0 ± 5.9	32.0 ± 9.6	31.3 ± 5.1
**Height (m)**	1.67 ± 0.07	1.66 ± 0.06	1.78 ± 0.07	1.77 ± 0.06
**Body mass (kg)**	64.2 ± 7.5	65.6 ± 11.4	86.0 ± 9.2	79.1 ± 10.2
**Body mass index (kg/m^2^)**	23.0 ± 3.5	23.7 ± 3.5	27.2 ± 3.1	25.2 ± 2.1

**Table 2:** Dietary intake data in men before (PRE), in the middle (MID) and after (POST) the 12-week diet period.

Parameters	Low-PRAL			Moderate-PRAL		
	PRE	MID	POST	PRE	MID	POST
PRAL (mEq/day)	23 ± 32	−41 ± 24 ^†††^	−37 ± 24 ^††^	8.1 ± 16	10 ± 12 ***	11 ± 17 **
IFV (g/day)	250 ± 140	900 ± 300	800 ± 380	300 ± 250	250 ± 250 ***	230 ± 100 *
Energy(kcal/day)	2670 ± 910	1930 ± 570 ^†^	1930 ± 520	2220 ± 630	2210 ± 650	2180 ± 690
Protein (g/kg/day)	1.5 ± 0.8	1.0 ± 0.3	1.1 ± 0.2	1.3 ± 0.5	1.4 ± 0.5	1.4 ± 0.4
CHO (g/kg/day)	3.4 ± 1.6	2.2 ± 1.0	2.5 ± 1.7	2.9 ± 0.9	3.2 ± 0.9	3.1 ± 0.9
Fat (g/kg/day)	1.2 ± 0.5	0.8 ± 0.3	0.8 ± 0.3	1.2 ± 0.4	1.2 ± 0.4	0.9 ± 0.4

CHO, carbohydrates; IFV, intake of fruits and vegetables. * *p* < 0.05, ** *p* < 0.01, *** *p* < 0.001 statistically significant difference between low- and moderate-PRAL at POST. ^†^ *p* < 0.05, ^††^ *p* < 0.01, ^†††^ *p* < 0.001 statistically significant difference between PRE and MID or PRE and POST in low-PRAL (two-way repeated measures ANOVA, a paired or independent *t*-test).

**Table 3:** Dietary intake data in women before (PRE), in the middle (MID) and after (POST) the 12-week diet period.

Parameters	Low-PRAL			Moderate-PRAL		
	PRE	MID	POST	PRE	MID	POST
PRAL (mEq/day)	−7.2 ± 18	−51 ± 19 ^†††^	−56 ± 40 ^††^	−2.9 ± 11	3.6 ± 11 ***	−0.8 ± 17 ***
IVF (g/day)	400 ± 200	930 ± 310	1070 ± 630	250 ± 80	210 ± 160 ***	260 ± 270 ***
Energy (kcal/day)	2010 ± 380	1880 ± 360	1860 ± 500	1900 ± 280	1990 ± 580	1870 ± 340
Protein (g/kg/day)	1.3 ± 0.5	1.1 ± 0.2	1.1 ± 0.3	1.2 ± 0.2	1.4 ± 0.4	1.1 ± 0.2
CHO (g/kg/day)	3.6 ± 0.8	3.6 ± 0.8	3.8 ± 1.3	3.6 ± 0.7	3.8 ± 1.6	3.2 ± 0.8
Fat (g/kg/day)	1.3 ± 0.3	1.1 ± 0.4	1.0 ± 0.8	1.1 ± 0.4	1.2 ± 0.5	1.0 ± 0.2

CHO, carbohydrates; IFV, intake of fruits and vegetables. *** *p* < 0.001 statistically significant difference between low- and moderate-PRAL at POST. ^††^ *p* < 0.01, ^†††^ *p* < 0.001 statistically significant difference between PRE and MID or PRE and POST in low-PRAL (two-way repeated measures ANOVA, a paired or independent *t*-test).

**Table 4:** Body composition before (PRE), and after (POST) the 12-week diet period in low-PRAL and moderate-PRAL diet groups.

Parameters	Men				Women			
	Low-PRAL		Mod-PRAL		Low-PRAL		Mod-PRAL	
	PRE	POST	PRE	POST	PRE	POST	PRE	POST
Body mass (kg)	85.5 ± 9.8	83.7 ± 9.5	79.2 ± 10.2	79.6 ± 9.8	64.3 ± 7.8	63.8 ± 7.9	67.0 ± 11.1	67.9 ± 11.5
Lean mass (kg)	61.5 ± 5.6	61.3 ± 5.2	56.1 ± 4.8	57.2 ± 5.8	41.2 ± 3.4	41.5 ± 2.6	40.9 ± 4.8	41.7 ± 4.9
Fat %	23.9 ± 7.4	22.0 ± 7.9	25.3 ± 6.9	23.8 ± 5.9	31.0 ± 7.0	30.4 ± 6.6	33.2 ± 9.3	33.8 ± 9.0
